# Construction and Comprehensive Analysis of a Molecular Association Network via lncRNA–miRNA–Disease–Drug–Protein Graph

**DOI:** 10.3390/cells8080866

**Published:** 2019-08-09

**Authors:** Zhen-Hao Guo, Hai-Cheng Yi, Zhu-Hong You

**Affiliations:** 1 The Xinjiang Technical Institute of Physics and Chemistry, Chinese Academy of Sciences, Urumqi 830011, China; 2 University of Chinese Academy of Sciences, Beijing 100049, China

**Keywords:** network biology, LINE, lncRNA, protein, miRNA, drug, disease

## Abstract

One key issue in the post-genomic era is how to systematically describe the associations between small molecule transcripts or translations inside cells. With the rapid development of high-throughput “omics” technologies, the achieved ability to detect and characterize molecules with other molecule targets opens the possibility of investigating the relationships between different molecules from a global perspective. In this article, a molecular association network (MAN) is constructed and comprehensively analyzed by integrating the associations among miRNA, lncRNA, protein, drug, and disease, in which any kind of potential associations can be predicted. More specifically, each node in MAN can be represented as a vector by combining two kinds of information including the attribute of the node itself (e.g., sequences of ncRNAs and proteins, semantics of diseases and molecular fingerprints of drugs) and the behavior of the node in the complex network (associations with other nodes). A random forest classifier is trained to classify and predict new interactions or associations between biomolecules. In the experiment, the proposed method achieved a superb performance with an area under curve (AUC) of 0.9735 under a five-fold cross-validation, which showed that the proposed method could provide new insight for exploration of the molecular mechanisms of disease and valuable clues for disease treatment.

## 1. Introduction

There are many types of biomolecules inside living cells that form multiple associated regulatory networks as pathways or direct participants to maintain a wide variety of life activities and key functions [1,2,3]. For instance, protein–protein interactions play a key role in numerous life processes and maintain many functions of normal cells. There is also growing evidence that ncRNAs are involved in cell growth and apoptosis, leading to numerous diseases. Therefore, predicting the potential associations between small molecule transcripts and compounds not only helps people to understand important cell activities at the molecular level, but is also significant for prevention, diagnosis, and treatment of disease, as well as genomic drug discovery [4,5]. In fact, it is unrealistic to verify the existence of association between such large-scale nodes one by one through biological experiments under the constraints of time and cost. In addition, the results of the experimental methods will be accompanied with higher false positives and false negatives due to various external factors [6]. Benefiting from the development of high-throughput technologies such as microarray, q-PCR, and yeast two-hybrid screens (Y2H) [7,8], construction of association prediction framework that provide a new viewpoint for gaining a holistic understanding in different fields will be possible based on the published online database such as LncRNADisease [9], HMDD [10], and STRING [11].

In recent years, several computational methods based on public data sets have been put forward successively and was carried on in practice to guide and support manual experiments [12]. These proposed methods can be roughly divided according to the research field, calculation model, calculation method, etc. The prediction model can be divided into several categories according to the different research objects and the typical representatives are as follows. In the field of protein–protein interaction (PPI), Wang et al. [13] regarded the protein sequence as a kind of natural language called Bio2Vec for feature extraction and discover the potential association by convolution neural network (CNN). In the field of the non-coding RNA (ncRNA)-protein interaction (RPI), Yi et al. proposed a robust deep learning framework for predicting interactions through evolutionary information [14]. In the field of drug-target interactions (DTI), Wang et al. predict the association between drug and target by rotation forest based on drug structure and protein sequence [15]. Through the different computational models, the prediction framework can be roughly divided into machine learning based methods, network based methods, and matrix decomposition based methods. You et al. proposed a novel model called PCA-EELM to predict protein–protein interactions by a machine learning model with only information of the protein sequence [16]. Chen et al. presented a network-based framework to predict miRNA-disease association by integrating known associations and the similarity of miRNAs and diseases respectively [17]. Li et al. transformed the problem of discovering undetected miRNA-disease association into the problem of adjacency matrix completion and proposed a prediction model called MCMDA [18]. Most of the existing computational models are based on direct associations or the characteristics of the research objects themselves to detect unknown relationships. Now, it is becoming more and more popular to explore potential associations through some intermediary. For example, by constructing a heterogeneous network of miRNA, lncRNA, and disease, Chen et al. took lncRNA as an intermediary to discover miRNA-disease associations through label propagation algorithms [19]. Peng et al. carried on CNN as the classifier to predict undetected microRNA (miRNA)-disease associations by capturing similarity in a three-layer network including miRNA, protein, and disease [20]. Researchers are gradually addressing this problem through an increasingly overall perspective, but to date there is still no predictive model that can discover the association of any node in the complete network within a cell.

**Figure 1:**
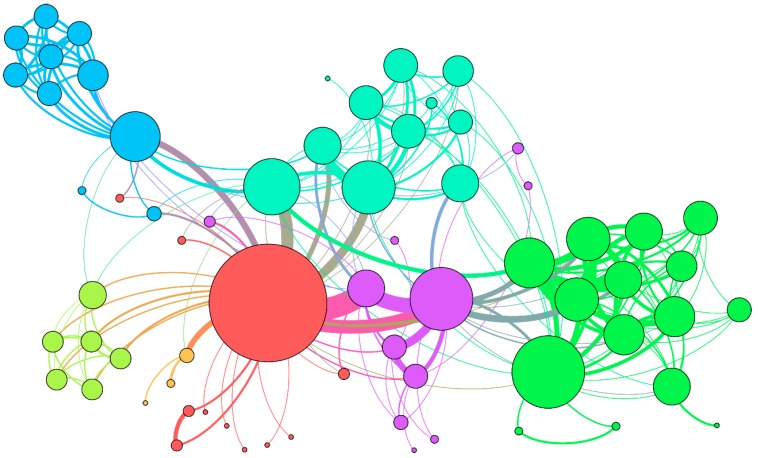
Example of the molecular association network. A different color represents different types molecule nodes and associations edges.

**Figure 2:**
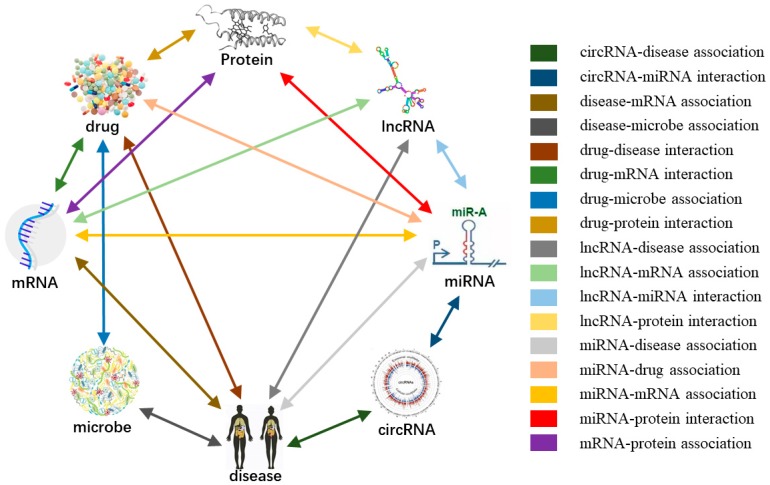
Systematic integration of multiple associations between biomolecules to construct the molecular association network.

In this study, we present a model to predict the relationship between any small molecules in a cell based on the node attribute and node behavior through a more systematic and comprehensive view. The complex associations network of biomolecules (as shown in Figure 1 and Figure 2) consists of two parts: Nodes (miRNA, long noncoding RNA (lncRNA), protein (target), drug, and disease) and edges (the relationships between nodes). Determining the edges between any two nodes in the whole complex network helps people to have a deep and comprehensive understanding of various life activities in living organisms from another micro perspective [21,22].

Firstly, nine kinds of molecular associations, such as miRNA-disease association, protein–protein interaction, lncRNA-disease associations, and drug-target interaction were collected to consider the relationship between each node and any other kind of node in a global way. After de-redundancy and repetition, five research objects such as miRNA, protein, and drug were obtained and co-combined to construct a complex heterogeneous network in an entire view at the cellular level. Secondly, each node can be represented in two ways. One is the node intrinsic attributes such as the sequence of ncRNA and protein, the molecular fingerprint of drug, and the semantics of disease. The other way is to represent nodes as vectors through network embedding based on the relationship between nodes. Thirdly, all known associations are treated as positive samples, and an equal number of unknown associations are randomly selected as negative samples, which together serve as a training set. Random forest is selected as a classifier for training verification and testing. The five-fold cross validation was adopted to evaluate the proposed method, and we also compared the performance of different types of features, classifiers, and previous methods. The results indicate that our method combined intrinsic attribute feature and behavior feature could achieve an effective and robust prediction performance. The construction of systematic and complex molecular association network offers a new view, which can help us better understand biology and disease pathologies. To the best of our knowledge, we are the first to construct a molecular association network (MAN) using associations between lncRNA, miRNA, disease, drug, and protein. We hope that this work will inspire more research on representational learning on biological networks.

## 2. Materials and Methods

### 2.1. Construction of the Molecular Association Network

In order to systematically and holistically establish the molecular association network, known associations between biological molecule transcripts (miRNA, lncRNA, and protein), diseases and drugs were downloaded from multiple databases. The details of the final experimental data obtained after performing the inclusion of identifier unification, de-redundancy, simplification, and deletion of the irrelevant items are shown in the following Table 1.

After aggregating the above database, we separately classified the different nodes to get the final statistics as shown in the following Table 2.

### 2.2. NcRNA and Protein Sequence

The sequences of miRNA, lncRNA, and protein are downloaded from miRbase [29], NONCODE [30], and STRING [11], respectively, to subsequently represent the attribute of the node. For the sake of simplicity, we chose to encode ncRNA sequences using a 64 (4 × 4 × 4) dimensional vector, in which each feature represents the normalized frequency of the corresponding 3-mer appearing in the RNA sequence (e.g., ACG, CAU, UUG). Inspired by the article of Shen et al. [31], in the process of protein sequence encoding, 20 amino acids are classified into four classes according to the polarity of the side chain including Ala, Val, Leu, Ile, Met, Phe, Trp, and Pro; Gly, Ser, Thr, Cys, Asn, Gln, and Tyr; Arg, Lys, and His; and Asp and Glu. Thus, each protein sequence can be represented by a 3-mer that is a 64 (4 × 4 × 4) dimensional vector and each dimension of the vector representing the normalized frequency of the corresponding 3-mer in the sequence.

### 2.3. Disease MeSH Descriptors and Directed Acyclic Graph

**Figure 3:**
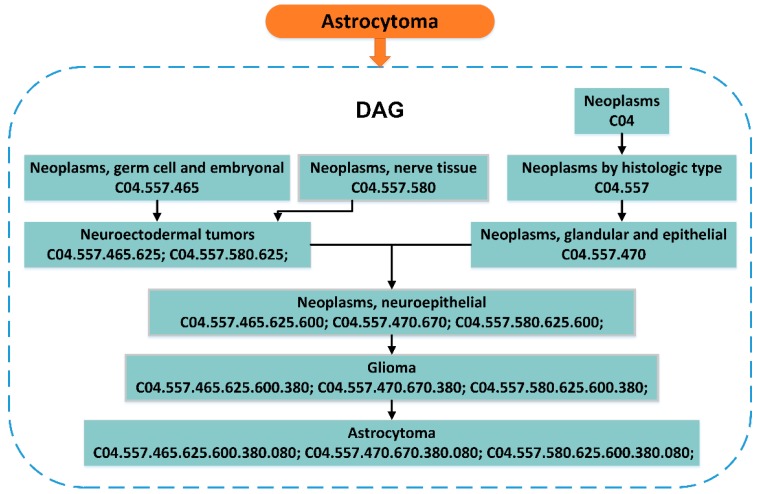
Construction of the Astrocytoma’s directed acyclic graph (DAG). Remove the last three digits of a Medical Subject Headings (MeSH) descriptor of the disease to get a new MeSH descriptor of a more abstracted disease. For example, Neoplasms, neuroepithelial, and the last three digits of its descriptor C04.557.465.625.600 are removed to get neuroectodermal tumors (C04.557.465.625).

The Medical Subject Headings (MeSH) is a comprehensive searchable control vocabulary, which is organized by the National Library of Medicine furnished a rigorous index for journal articles and books in the life sciences. The top-level categories in the MeSH descriptor hierarchy are: Anatomy (A), Organisms (B), Diseases (C), Chemicals Drugs (D), and so on. In this system, each disease can be represented by a directed acyclic graph (DAG) generated through its MeSH, accurately and objectively describe its own characteristics. The details to describe the disease with DAG is as follows: DAG(D) = (D, N(D), E(D)), N(D) is the set of points that contains all the diseases in the DAG(D). E(D) is the set of edges that contains all relationships between nodes in the DAG(D). An example of the disease Astrocytoma’s DAG is as follows in Figure 3.

For the diseases that are included in MeSH, the semantic similarity that is calculated by means of DAG can be chosen to represent the disease according to the previous literature [32]. The semantic similarity between different diseases can be defined as follows. In DAG of disease *D*, the contribution of any ancestral disease *t* to disease *D* is as the formula:

(1) $$\{\begin{array}{c} D_{D}\left(t\right)=1\;if\;t=D\\ D_{D}\left(t\right)=\max\left\{\Delta *D1_{D}\left(t^{\prime }\right)|t^{\prime }\in children\;of\;t\right\}\;if\;t\neq D \end{array}$$

Δ is the semantic contribution factor and equals to 0.5 according to the literature mentioned above. The contribution of disease *D* to itself is 1 and the contribution of other nodes to D will be attenuated due to Δ. Based on Equation (1), we can obtain the sum of the contributions of all diseases in DAG to *D*:

(2) $$DV\left(D\right)=\Sigma_{t\in N\left(D\right)}D_{D}\left(t\right)$$

Similar to the Jaccard similarity coefficient, the semantic similarity between the diseases *i* and *j* can be calculated by the following formula:

(3) $$S1\left(i,j\right)=\frac{\sum_{t\in N\left(i\right)\cap^{\;}N\left(j\right)}\left(D_{i}\left(t\right)+D_{j}\left(t\right)\right)}{DV\left(i\right)+DV\left(j\right)}$$

### 2.4. Drug Molecular Fingerprint

The smiles of drugs were downloaded from DrugBank and then transformed into the corresponding Morgan molecular fingerprint by the python package.

### 2.5. Stacked Autoencoder

In order to reduce noise and normalize attribute information to a uniform dimension, a stacked autoencoder was employed to obtain a suitable subspace from the original feature space. Stacked Autoencoder (SAE) can be divided into two parts: The encoder that encodes the input data into corresponding representation *h* and the decoder that reconstructs an approximation *x̂* from the hidden representation *h*.

(4) $$h=f\left(x\right)≔S_{f}\left(Wx+p\right)$$

(5) $$y=g\left(h\right)≔S_{g}\left(W^{\prime }x+q\right)$$

Here, we choose the ReLU function as the activation function:

(6) $$S_{f}\left(t\right)=S_{g}\left(t\right)=max\left(0,Wt+b\right)$$

### 2.6. Node Representation

In the entire biomolecular network, each node can be represented by the node intrinsic attributes and behavior. The attributes of the node itself can be the sequence of ncRNA, protein, the semantics of the disease, and the molecular fingerprint of the drug. The node behavior can be considered as a representation of the relationship between nodes. More specifically, in this work, we chose a method of network embedding called LINE [33] to globally represent the behavior of nodes in the entire network and the flow of information directly or latently with other nodes.

For large-scale networks, some existing network representation learning algorithms require complex computational complexity. Recently, some methods of large-scale networks either use indirect methods to reduce computational complexity or lack an explicit objective function (DeepWalk). LINE defines two similarity relationships, including the first-order proximity and the second-order proximity. The first-order similarity is defined as the node connection relationship (local feature) in the network, and the second-order similarity is defined as the common neighbor node (global feature) of the nodes that are not directly connected as a supplement to the first-order similarity. In this work, we used the network representation model LINE to learn how to represent the relationships between each node and other nodes in the entire network. In this way, an undirected edge can be considered as two directed edges with opposite directions and equal weights. The second-order proximity assumes that vertices sharing many connections to other vertices are similar to each other. For each directed edge (*i*, *j*), the probability of “context” $v_{j}$ generated by vertex $v_{i}$ is defined as:

(7) $$p_{2}\left(v_{j}|v_{i}\right)=\frac{\exp\left({\vec{u}}_{j}^{T}\cdot {\vec{u}}_{i}\right)}{\sum_{k=1}^{\left|V\right|}\exp\left({\vec{u}}_{k}^{T}\cdot {\vec{u}}_{i}\right)}$$

Therefore, we minimize the following objective functions:

(8) $$O_{2}=\underset{i\in V}{\sum }\lambda_{i}d\left({\hat{P}}_{2}\left(\cdot |v_{i}\right),p_{2}\left(\cdot |v_{i}\right)\right).$$

The empirical distribution ${\hat{P}}_{2}\left(\cdot |v_{i}\right)$ is defined as:

(9) $${\hat{P}}_{2}(v_{j}|v_{i})=\frac{w_{ij}}{d_{i}}$$

For the sake of simplicity, $\lambda_{i}$ is set to the degree of the vertex *i*, i.e., $\lambda_{i}=d_{i}$. Here Kullback-Leibler (KL) divergence is used as the function of distance. After some constants are omitted, the loss function can be simplified as the following form:

(10) $$O_{2}=-\underset{\left(i,j\right)\in E}{\sum }w_{i,j}\log p_{2}\left(v_{j}|v_{i}\right)$$

## 3. Results and Discussion

### 3.1. Evaluate the Five-Fold Cross Validation Performance of Our Method

For five-fold cross validation, the entire data set was randomly divided into five subsets of equal size, one subset was treated as the test set in turn, and the remaining four subsets were used as the training sets to construct the classifier. Note that at the time of each cross validation, only the currently training set, i.e., 80% of the total edges, would be embedding as the behavior of the node, which avoids the leakage of test information. Although the above operations may cause some of the nodes originally in the network to become isolated, i.e., degree with 0 and these nodes may also lack attribute information at the same time. This situation can better simulate the real environment to provide support and assistance for the exploration of unknown fields by researchers through manual experiments.

**Figure 4:**
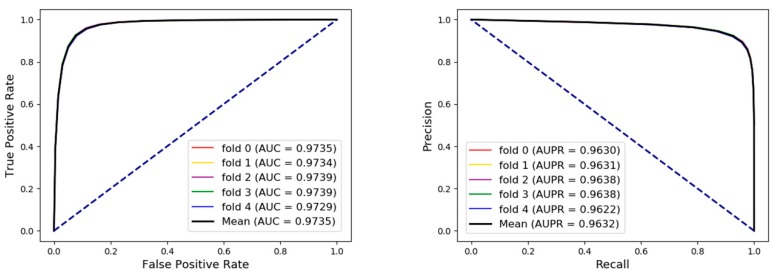
The receiver operating characteristic curves (ROCs), area under curves (AUCs), Precision-Recall Curves (PRs), and area under PRs (AUPRs) of our method under five-fold cross validation on the whole dataset.

A range of broader evaluation criteria including accuracy (Acc.), sensitivity (Sen.), specificity (Spec.), precision (Prec.), and Matthews Correlation Coefficient (MCC) were utilized to evaluate the proposed model more comprehensively and fairly. As shown in Table 3 and Figure 4, the results of average Acc., Sen., Spec., Prec., MCC, and area under curve (AUC) of 92.38%, 92.61%, 92.14%, 92.18%, 84.76%, and 97.35% when the proposed framework was applied to predict arbitrary associations in the whole network. Receiver operating characteristic curve (ROC) is a commonly used standard for evaluating models. Area under curve (AUC) is the area of graph that is surround by the ROC, the abscissa false positive rate (FPR), and the ordinate true positive rate (TPR). We also draw the ROC and calculated AUC to visually evaluate our proposed model at the same time under five-fold cross validation. Precision-Recall (PR) curve, whose abscissa is the recall and ordinate is the precision, was applied to evaluate the model from another angle. In conclusion, our method obtained an AUC of 0.9735 and AUPR (area under PR) that indicated that the proposed method combined two types of feature had an excellent ability to identify positive and negative samples. The higher AUC and AUPR indicated our method had a strong predictive performance and the lower variance of the results showed the proposed model was stable and robust.

### 3.2. Comparison of Different Feature Extraction Methods

**Figure 5:**
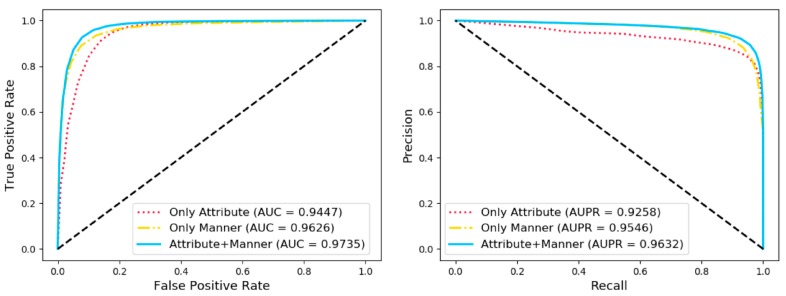
Comparison with different features under five-fold cross validation.

As mentioned above, each node in the biomolecular network within the cell can be represented by two kinds of information including attribute information and behavior information. In order to evaluate the impact of each type of information on the final classification effect, we respectively utilized the information of attribute, the information of behavior and the combination of the above two to represent the node under the extensive evaluation criteria in five-fold cross validation. As shown in Table 4 and Figure 5, the average of ROC and PR under five-fold cross validation is reported. A variety of evaluation criteria as shown in the table below indicated that the node representation vectors combined with the two kinds of information has more outstanding expressiveness.

### 3.3. Comparison of Different Classifiers

**Figure 6:**
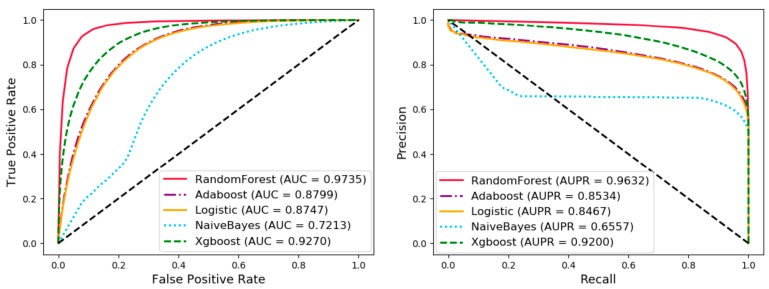
Comparison with random forest, Adaboost, logistic regression, naive Bayes, and XGBoost under five-fold cross validation.

In order to evaluate the performance of the classifier, we compared random forest with Adaboost, logistic regression, naïve Bayes, and XGBoost under five cross-validation in various evaluation criteria. Under the control variable method, the various settings of the experiment are the same except the classifier, which makes the comparison of experimental results fairer and more credible. The results are shown in Table 5 and Figure 6. The difference in the effect of different classifiers may be caused by the following factors: (1) Naive Bayes can get better results when the properties of the sample are independent of each other. In this experiment, there are cases where the attributes are not independent and cross-joining together affects the final classification effect. (2) Logistic regression is essentially a linear classifier whose performance is limited by the distribution of the data and did not perform well in this article. (3) The parameters of all classifiers are default values, which may cause Adaboost and XGBoost to have under-fitting or over-fitting on this task.

### 3.4. Additional Comparison Experiment for lncRNA-Disease Association Prediction

In order to compare with traditional methods that focus on single or isolated objects, the lncRNA-disease association prediction was chosen to perform this comparison experiment because of the serious lack of node attribute information. After processing the data, 1263 independent lncRNA-disease association pairs were obtained including 345 different lncRNAs and 295 different diseases. The sequence of each lncRNA was determined when the experimental materials were collected. However, among all diseases associated with lncRNA, only 76 of 295 diseases were able to obtain attribute information by constructing DAG to produce similarity with other diseases. The pairs, which include both two kinds of information only took possession of 259 in 1263 associations.

**Figure 7:**
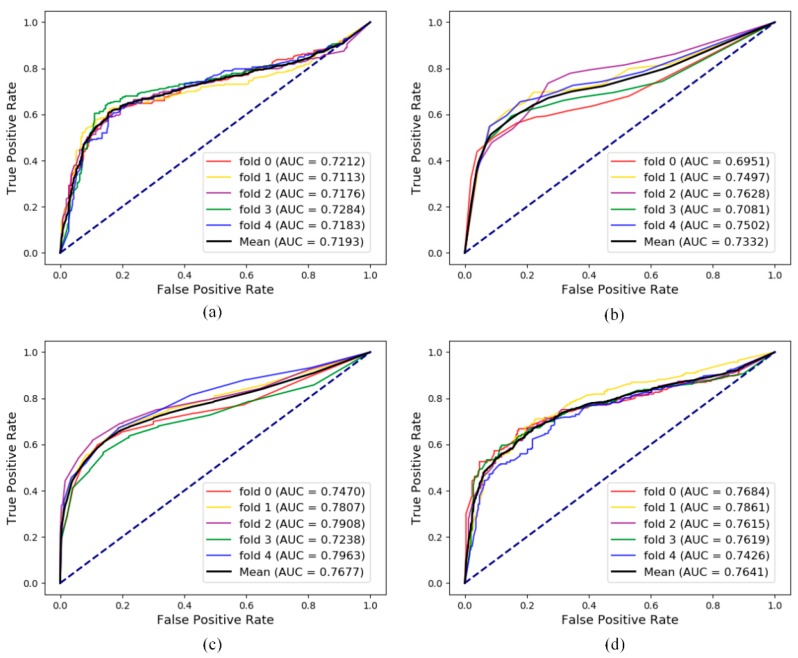
Comparison with different feature for lncRNA-disease association prediction. (**a**) The five-fold cross-validation performance using only the attribute feature; (**b**) the five-fold cross-validation performance using the Gaussian profile kernel similarity feature; (**c**) the five-fold cross-validation performance using only the behavior feature generated by LINE using other eight associations in the whole MAN and training data in lncRNA-disease data; and (**d**) the five-fold cross-validation performance using behavior feature generated by LINE using other eight associations in the whole MAN.

Figure 7a shows results of the link prediction under five-fold cross validation with pure attribute information as the characteristics of the node. It can be treated as a baseline. Figure 7b shows the results of link prediction based on the feature combined attribute feature with previous isolated embedding (behavior) feature. That is, 80% lncRNA-disease associations were utilized to construct the adjacency matrix for generating Gaussian profile kernel similarity in each fold cross validation [34]. Figure 7c show the results of global embedding, that is, 80% of the lncRNA-disease associations and all the other eight kinds of associations were processed by LINE in each cross-validation. Obviously, after combining the global behavior information, the performance of prediction in lncRNA-disease association can be greatly improved. It also proves that the cell is a complete unit of life, and the interaction of biomolecules in the cell together maintains the normal conduct of life activities. Figure 7d show the results of a special graph embedding strategy inspired by Chen et al. [35]. Eight other associations in the whole network besides lncRNA-disease pairs were processed by LINE in each fold cross-validation. LncRNA-disease pair vectors combined attribute feature and behavior feature were sent into random forest classifiers for prediction and evaluation. Inspired by experiment 7d, we did a case study based on lncRNA NONHSAT017462.2. The lncRNADisease database (remove all lncRNA NONHSAT017462.2 association pairs) was used as the training data set to construct the model. The pairs between lncRNA NONHSAT017462.2 and all diseases were predicted by the model. Furthermore, we converted lncRNA NONHSAT017462.2 to its alias h19 and did cross-dataset validation in another dataset MNDR 2.0 [36]. The verification of Top-20 prediction is as follows in Table 6.

### 3.5. Analysis Based on a Specific miRNA, lncRNA, and Protein

**Figure 8:**
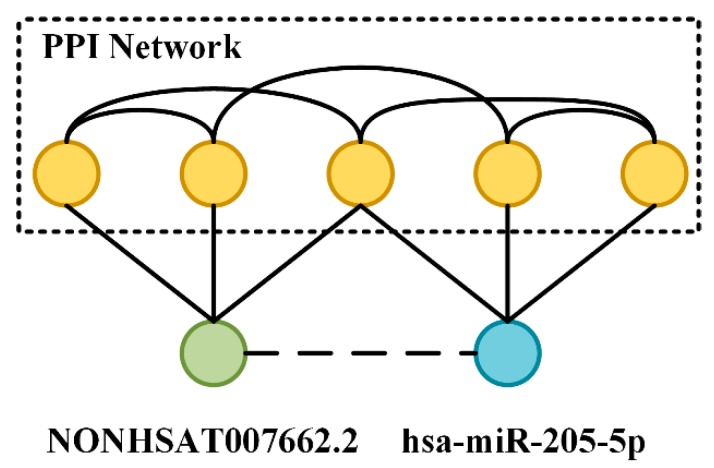
NONHSAT007662.2/hsa-miR-205-5p interaction and their PPI network.

We tried to analyze the flow of information in the molecular association network (MAN) based on a specific miRNA, lncRNA, and protein, which makes the model more interpretable. Two pairs of lncRNA–miRNA interactions were selected to perform this experiment. Firstly, each lncRNA and miRNA-associated protein was queried and aggregated. Then the obtained proteins were constructed into a PPI network. LncRNA and miRNA were directly connected and therefore were each other’s first-order neighbors. The process of a message transmission through the PPI network as an intermediary makes lncRNA and miRNA each other’s high-order neighbors, although the intersection of the second-order neighbors was not large, but the third-order neighbors and their associations were very rich. The specific lncRNA–miRNA interactions and associated proteins are shown in the following Table 7 and Figure 8.

## 4. Conclusions

Accumulating evidence demonstrated the superiority of link prediction based on massive data through machine learning models, which not only served as an addition to manual experiments, but also provided researchers with an overall and macro insight into the interactions between intracellular molecules. In this article, we proposed a novel framework based on five different kinds of nodes and nine different kinds of relationships to detect any potential associations between arbitrary research objects in the whole network. Each node could be represented as a vector by two kinds of information including node attribute and node behavior. For attribute information, ncRNA and protein could be encoded into 64-dimensional vectors by the method of k-mer, in which each feature represents the normalized frequency of the corresponding 3-mer appearing in the RNA or protein sequence. The characteristics of the disease and the drug could be represented by their own semantic and molecular structure and transformed into 64-dimensional vectors through the function of feature selection and transformation in SAE. For behavior information, the relationships of each node with others could be abstracted by the network embedding method LINE. Combined with the above two kinds of feature, each node could be represented by a 128-dimensional vector and random forest classifier was chosen to carry out prediction tasks. The experimental results showed that our method could achieve outstanding performance. The construction of the molecular association network (MAN) in human cells, offers a new systematic view on understanding complex life activities and diseases.

## Figures and Tables

**Table 1:** The details of nine kinds of associations in the molecular association network (MAN).

Relationship Type	Database	Number of Associations
miRNA–lncRNA	lncRNASNP2 [23]	8374
miRNA–disease	HMDD [10]	16,427
miRNA–protein	miRTarBase [24]	4944
lncRNA–disease	LncRNADisease [9] lncRNASNP2 [23]	1264
lncRNA–protein Protein–disease Drug–protein Drug–disease Protein–protein	LncRNA2Target [25] DisGeNET [26] DrugBank [27] CTD [28] STRING [11]	690 25,087 11,107 18,416 19,237
Total	N/A	105,546

**Table 2:** The amount of five types of nodes in the molecular association network (MAN).

Node	Number of Nodes
Disease	2062
LncRNA	769
MiRNA	1023
Protein	1649
Drug	1025
Total	6528

**Table 3:** Five-Fold cross-validation results performed by our method.

Fold	Acc. (%)	Sen. (%)	Spec. (%)	Prec. (%)	MCC (%)	AUC (%)
0	92.25	92.68	91.82	91.89	84.51	97.35
1	92.43	92.52	92.35	92.36	84.87	97.34
2	92.49	92.84	92.13	92.19	84.98	97.39
3	92.58	92.75	92.42	92.44	85.16	97.39
4	92.13	92.28	91.98	92.01	84.26	97.29
**Average**	**92.38** **± 0.18**	**92.61** **± 0.22**	**92.14** **± 0.25**	**92.18** **± 0.23**	**84.76** **± 0.37**	**97.35** **± 0.04**

**Table 4:** Comparison of different features.

Feature	Acc. (%)	Sen. (%)	Spec. (%)	Prec. (%)	MCC (%)	AUC (%)
Attribute	88.62 ± 0.14	91.48 ± 0.13	85.76 ± 0.2	86.53 ± 0.17	77.37 ± 0.28	94.47 ± 0.11
Behavior	90.7 ± 0.14	88.84 ± 0.15	92.56 ± 0.19	92.27 ± 0.19	81.45 ± 0.29	96.26 ± 0.05
**Both**	**92.38** **± 0.18**	**92.61** **± 0.22**	**92.14** **± 0.25**	**92.18** **± 0.23**	**84.76** **± 0.37**	**97.35** **± 0.04**

**Table 5:** Comparison of different classifiers.

Classifier	Acc. (%)	Sen. (%)	Spec. (%)	Prec. (%)	MCC (%)	AUC (%)
Adaboost	80.03 ± 0.29	80.91 ± 0.3	79.14 ± 0.43	79.51 ± 0.36	60.07 ± 0.58	87.99 ± 0.28
Logistic	79.92 ± 0.29	82.78 ± 0.29	77.06 ± 0.49	78.3 ± 0.37	59.94 ± 0.57	87.47 ± 0.26
Naive Bayes	55.93 ± 0.15	24.83 ± 0.24	87.04 ± 0.32	65.7 ± 0.5	15.15 ± 0.41	72.13 ± 0.34
XGBoost	84.37 ± 1.3	82.89 ± 2.96	85.85 ± 0.56	85.42 ± 0.37	68.8 ± 2.58	92.7 ± 0.66
**Random Forest**	**92.38** **± 0.18**	**92.61** **± 0.22**	**92.14** **± 0.25**	**92.18** **± 0.23**	**84.76** **± 0.37**	**97.35** **± 0.04**

**Table 6:** Top-20 prediction verified in the lncRNADisease and MNDR 2.0 databases.

Number	Disease Name	Probability	Evidence
1	Melanoma	0.85	LncRNADisease
2	Cervical cancer	0.85	LncRNADisease
3	Rheumatoid arthritis	0.85	MNDR 2.0
4	Hepatocellular carcinoma	0.85	MNDR 2.0, LncRNADisease
5	Myelodysplastic syndrome	0.846551724	Unconfirmed
6	Schizophrenia	0.842857143	Unconfirmed
7	Chronic lymphocytic leukemia	0.840909091	Unconfirmed
8	Diffuse large b-cell lymphoma	0.837460815	Unconfirmed
9	Cardiac hypertrophy	0.833766234	Unconfirmed
10	Digeorge syndrome	0.8	Unconfirmed
11	Multiple sclerosis	0.8	Unconfirmed
12	Acute promyelocytic leukemia	0.786551724	Unconfirmed
13	Autism spectrum disorder	0.75	Unconfirmed
14	Colorectal cancer	0.75	MNDR 2.0, LncRNADisease
15	Osteosarcoma	0.75	MNDR 2.0, LncRNADisease
16	Squamous cell carcinoma	0.75	MNDR 2.0, LncRNADisease
17	Atherosclerosis	0.75	LncRNADisease
18	Glioblastoma	0.75	LncRNADisease
19	Pituitary adenoma	0.75	LncRNADisease
20	Pre-eclampsia	0.75	LncRNADisease

**Table 7:** Two specific lncRNA–miRNA pairs and their protein–protein interaction (PPI) network constructed by associated proteins.

LncRNA–miRNA Pairs	Associated Proteins Respectively	PPI Network Edges
NONHSAT007662.2/hsa-miR-205-5p	70/27	1066
NONHSAT017460.2/hsa-miR-148a-3p	74/28	877
